# Development of A Surgery‐Related Pressure Injury Risk Assessment Scale (SURPIRAS): A Methodological Study

**DOI:** 10.1111/jocn.17765

**Published:** 2025-04-08

**Authors:** Sema Konateke, Şükriye İlkay Güner

**Affiliations:** ^1^ Department of Surgical Nursing, Faculty of Health Sciences Gaziantep University Gaziantep Türkiye

**Keywords:** nursing, operating room, pressure injury, risk assessment, scale, surgery related

## Abstract

**Aim:**

To develop the Surgery‐Related Pressure Injury Risk Assessment Scale (SURPIRAS) and conduct validity and reliability analyses.

**Design:**

A methodological study.

**Methods:**

Data were collected from 200 patients with “Patient Identification Form”, “Postoperative Patient Assessment Form”, “SURPIRAS”, “Risk Assessment Scale for Injuries Related to Surgical Position (ELPO)” and “Perioperative Pressure Injury Risk Assessment Scale (Munro Scale)”. Number, percentage and mean, standard deviation were calculated in descriptive statistics. Intraclass correlation coefficient was used to evaluate the validity and reliability of the scale to determine content validity, criterion‐related validity and interobserver agreement. The scale's cut‐off point, sensitivity and selectivity values were calculated. The study was prepared according to the STARD publication checklist, following EQUATOR guidelines.

**Results:**

Pressure injuries occurred in 20.5% of the patients. The total score of the SURPIRAS was found to be statistically significant in terms of postoperative pressure injury development. A positive correlation was found between the developed scale and ELPO and Munro Scale. The strength of this correlation is higher between SURPIRAS and Munro Scale. The cut‐off point for the SURPIRAS was determined as 27, and patients with a total score of 28 points and above were classified as high risk.

**Conclusion:**

It was determined that the SURPIRAS is a valid and reliable measurement tool in determining the risk of pressure injury in surgical patients in the Turkish population.

**Relevance to Clinical Practice:**

The first thing to do to prevent pressure injuries in surgical patients is risk assessment. The study provides a new contribution to the literature because it is the development of a risk assessment scale.

**Patient or Public Contribution:**

Patients were included in the study after being informed about the study before surgery and giving written and verbal consent. Patients were monitored for pressure injury by the researchers after surgery.

**Summary**

- Surgery‐related pressure injury occurs in the first 48–72 h following surgery.
- Pressure injury risk assessment should be performed using risk assessment scales with proven validity and reliability.
- SURPIRAS is a new measurement tool for determining the risk of surgery‐related pressure injury in adults with proven validity and reliability in the Turkish population.

## 1 Introduction

Pressure injury is defined as localised damage due to disruption of circulation in the skin and/or deep tissues caused by continuous and prolonged pressure and shearing forces (EPUAP/NPUAP/PPPIA 2019; Karg et al. 2019; Lei et al. 2022). Many different terms are used in the literature for this developing situation. These terms are pressure wound, pressure injury, decubitus and pressure ulcer (Engels et al. 2016; Kottner et al. 2020; Konateke 2021). These terms are often used in the same meaning. However, there are differences in their definitions and effects. These terms do not accurately describe a “pressure injury”. Pressure injury refers to localised tissue damage to the skin and underlying tissues caused by prolonged pressure, usually exacerbated by cutting forces. In contrast, pressure injuries encompass a broader spectrum of tissue damage, including those that have not yet progressed to ulceration (Chittoria et al. 2024). This distinction is crucial for clinical assessment and management. The NPUAP (2014) guideline states that injury to tissues can occur without ulceration. Therefore, the term was updated as “pressure injury” (Edsberg et al. 2016). Since some terms used in health services have the same spelling (such as intravenous‐IV and Stage‐IV), the numerical representation was changed. It was decided to use Arabic numerals instead of Roman numerals for staging pressure injuries. This may reduce confusion among health professionals (Association News 2022). In this definition, a separate visualised staging representation was also made for people with light and dark skin (Edsberg et al. 2016). Additional definitions of pressure injuries have also been added, such as medical device‐related pressure injuries and mucosal membrane pressure injuries (Association News 2022). These advances help to effectively diagnose pressure injury and provide standardised, evidence‐based care (Pearce 2024).

Pressure injuries were defined as a geriatric problem with significant morbidity and negative consequences towards the end of the twentieth century. Later, with the increase in chronic diseases and the emergence of surgery‐related injuries, treatment and management have increased in this field (Levine 2024). It is seen that many leading scientific studies in the field of pressure injury have been published in the last 15–20 years. Although it is reported that pressure injury can be easily defined, it is also stated that there are still difficulties in diagnosis (Stewart et al. 2022).

Surgery‐related pressure injury was first introduced by Hick in the 1970s. In 1971, Hicks examined the development of pressure injury in patients undergoing surgical intervention and found that pressure injury developed in 13% of patients whose operation lasted longer than 2 h. Therefore, he stated that each patient who underwent surgery should be evaluated, especially in the areas under pressure (Yüksel and Kandemir 2022).

## 2 Background

The risks of perioperative pressure injuries are quite high in pressure injuries that develop within 48–72 h after surgery (Konateke 2021). Pressure injury may develop within the first 24 h postoperatively (Yoshimura et al. 2018, 2020) or within the first 72 h (Khong et al. 2020; Konateke 2021; Tura et al. 2023). Surgery‐related pressure injuries are the most common type of hospital‐acquired pressure injuries (Fred et al. 2012; Engels et al. 2016). The incidence of surgical pressure ulcers in studies varies according to the type of surgery and patient population (Peng et al. 2024). The incidence of surgery‐related pressure injury ranges from 1.3% to 54.8% (Webster et al. 2015; Kim et al. 2018; Eberhardt et al. 2021; Konateke 2021). In Türkiye, this rate is between 8.9% and 40.4% (Soyer and Özbayır 2018; Celik et al. 2019; Kandemir et al. 2022). In a systematic analysis, the incidence of intraoperative pressure injury was reported to be 15% (Chen et al. 2012).

Despite advances in healthcare and innovative developments in treatment and care, intraoperative pressure injury is still a major problem today (Tura et al. 2023). Pressure injuries that develop during the intraoperative period are an important condition that should be considered among hospital‐acquired pressure injuries (EPUAP/NPUAP/PPPIA 2019; Tang et al. 2021). It has been reported that 30% of the discharged surgical patients are re‐hospitalised within the first 30 days due to pressure injury. It has also been reported that the mortality rate is higher in patients hospitalised due to pressure injury after surgery (Webster et al. 2015). It is known that surgical pressure injury negatively affects postoperative recovery (Tang et al. 2021) and increases the duration of hospitalisation by an average of 10 days (Berlowitz et al. 2011; Tang et al. 2021). Health care costs increase by 44% due to prolonged hospitalisation (Spector et al. 2016). The estimated cost of treating patients with pressure injuries ranges between $75 and 150 million U.S. annually (Tang et al. 2021). Many perioperative risk factors accelerate the development of pressure injury depending on the surgical intervention (Konateke 2021). Patient‐specific risk factors include high or low body mass index (BMI), comorbidities, malnutrition, low haemoglobin, while surgery‐specific risk factors include surgical position, the effect of pressure, friction and shear forces on the points in contact with the operating table, hypothermia, type of surgery and anaesthesia, duration of surgery, blood loss, use of heart‐lung machine, etc., which increase the risk of pressure injury (Rao et al. 2016; de Oliveira et al. 2017; Peixoto et al. 2019).

Pressure injuries require a multidisciplinary team approach and can be prevented with appropriate interventions (Engels et al. 2016). Interventions for the prevention of pressure injury are aimed at reducing or eliminating the modifiable risks (Webster et al. 2015; Aloweni et al. 2019). Evidence‐based guidelines state that the first step to prevent pressure injury is risk assessment (Engels et al. 2016). The identified risk factors should be determined objectively with risk diagnostic tools, and high‐risk patients should be evaluated primarily (EPUAP/NPUAP/PPPIA 2014:^2019^). Risk assessment should start at the patient's admission to hospital. Prevention strategies should be implemented by identifying patients at risk (Creehan and Black 2022; Yüksel and Kandemir 2022).

Surgery‐related pressure injury risk assessment tools are “Risk Assessment Scale for Injuries Related to Surgical Position (ELPO)”, “Munro Scale”, “Scott Triggers Scale”, “3S Intraoperative Pressure Injury Risk Assessment Scale” (Konateke 2021). When these scales were examined, it was determined that there were scale items that took a long time to evaluate, were not objective and could not be completed quickly by surgical team members. Therefore, there was a need to develop a new risk assessment tool that determines the risk of pressure injury that may develop related to surgery in our country, is simple to use, can be easily applied by operating room nurses and surgical team members, does not require much time to perform, is objective and does not contain items that are open to interpretation.

## 3 Methods

### 3.1 Aim

The aim of this study was to develop the Surgery‐Related Pressure Injury Risk Assessment Scale (SURPIRAS) and to conduct a validity and reliability study of SURPIRAS.

### 3.2 Research Questions

- Is this new scale (SURPIRAS) developed by the researchers a valid assessment tool in the Turkish population?
- Is this new scale (SURPIRAS) developed by the researchers a reliable assessment tool in the Turkish population?

### 3.3 Study Design

This study was conducted methodologically. The study was conducted in the Central Operating Room of a university hospital in Gaziantep, Türkiye between October 2023 and May 2024. Surgery‐Related Pressure Injury Risk Assessment Scale was prepared according to the Guidelines for reporting diagnostic/prognostic studies (STARD 2015) checklist, following EQUATOR guidelines (https://www.equator‐network.org/reporting‐guidelines/stard/) (File S1).

### 3.4 Participants and Study Sample

The population of the study consisted of patients who underwent surgery in the Operating Room of the hospital between the dates of the study. The risk of surgery‐related pressure injury was evaluated when calculating the sample size. For the risk of surgery‐related pressure injury predicted in the literature (Kandemir et al. 2022), the minimum number of patients to be sampled at 5% certainty and a 95% confidence interval for the risk of surgery‐related pressure injury predicted in the literature was determined as 172 (http://sampsize.sourceforge.net/iface/). A total of 200 patients who met the inclusion criteria were included in the sample.

Patients who voluntarily agreed to participate in the study were 18 years of age or older, literate, had no cognitive and communication problems that prevented the collection of study data and underwent elective surgery were included in the study. Patients who had pressure injury on admission to the operating room, who had an obstacle to the control of the areas under pressure after surgery (movement restriction, etc.), who had an obstacle to tympanic body temperature measurement (ear surgeries, etc.) and who underwent emergency surgery were excluded from the study.

### 3.5 Data Collection Tools

In the study, data were collected with the “Patient Descriptive Characteristics Form”, “Postoperative Patient Evaluation Form”, “SURPIRAS”, “ELPO” and “Munro Scale”.

#### 3.5.1 Patient Identifying Characteristics Form

This form was created by the researchers following a review of the literature. It consisted of questions about the patient's sociodemographic characteristics, chronic disease status, smoking, preoperative skin assessment, preoperative pain status, whether position changes were made during surgery, whether blood transfusions were made and the support surface used on the operating table (Kandemir et al. 2022). After the literature review, this form was sent to 3 experts in the field and their opinions were obtained. After the expert opinion, it was revised and finalised.

#### 3.5.2 Postoperative Patient Evaluation Form

The form was created by the researchers to determine the status and stage of postoperative pressure injury and the regions where the pressure injury developed. The NPUAP (2014) pressure injury staging system was taken into consideration during staging (Edsberg et al. 2016). The form included questions about the patient's pain in the areas under pressure, skin condition, skin colour, skin hydration, nerve injury findings (loss of sensation, localised pain, muscle weakness) and eye injury findings (stinging sensation, dryness, blurred vision). The patient's pain was questioned with the Numeric Rating Scale (NRS). This form was presented to 3 experts in the field and revised in line with their suggestions.

#### 3.5.3 Surgery‐Related Pressure Injury Risk Assessment Scale (SURPIRAS)

“SURPIRAS” was developed by the researchers and includes risk factors such as age, body mass index (BMI), ASA score, type of anaesthesia, duration of surgery, amount of bleeding, wetness in areas under pressure, change in position, use of support surfaces, development of hypothermia, use of heating devices, change in blood pressure and comorbidity. The lowest score obtained from the scale is 13 and the highest score is 52.

#### 3.5.4 Risk Assessment Scale for Injuries Related to Surgical Position (ELPO)

This scale was developed by Lopes et al. (2016) and the Turkish validity and reliability study was conducted by Sengul et al. (2022). The total score of the scale is between 7 and 35. If the patient's total scale score is 19 and below, the risk of pressure injury due to surgical positioning is classified as low risk, and if it is 20 and above, it is classified as high risk (Sengul et al. 2022).

#### 3.5.5 Munro Scale (Cassendra Munro's Pressure Ulcer Risk Assessment Scale)

The scale was developed by Munro (2010) and a Turkish validity and reliability study was conducted by Gül et al. (2021). The scale uses a cumulative total score to assess preoperative, intraoperative and postoperative pressure injury risk factors. According to the total score, it is classified as 15 points (low risk), 16–28 points (moderate risk) or 29 and above points (high risk) (Haisley et al. 2020; Gül et al. 2021).

### 3.6 Study Flow Chart

**FIGURE 1:**
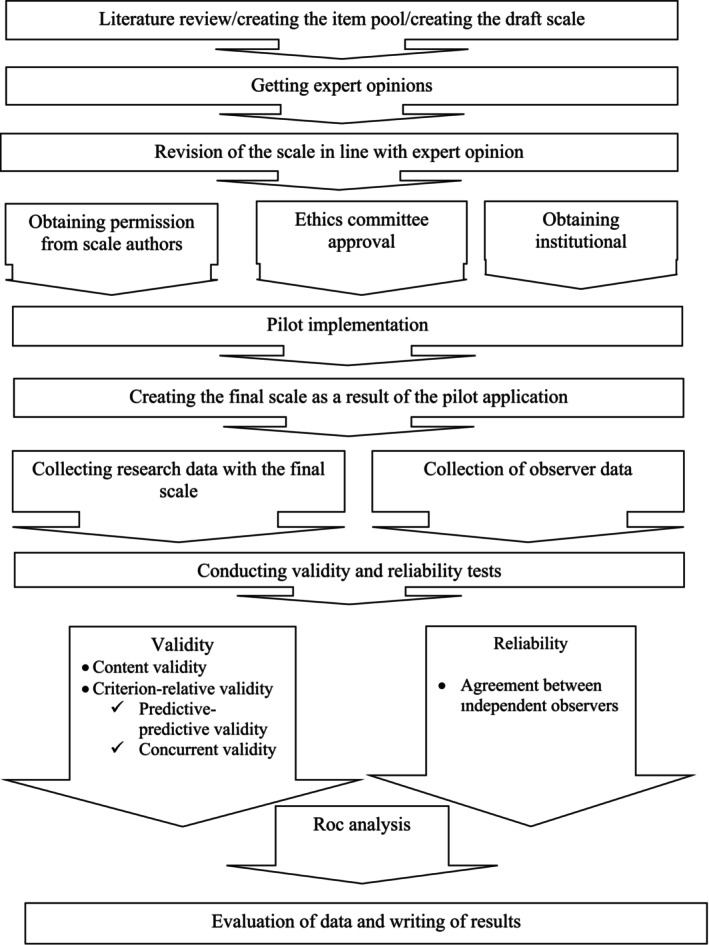
Flow chart.

The flow chart of the research is given below (Figure 1).

#### 3.6.1 Creating the Item Pool and Draft Scale

A detailed literature review on surgery‐related pressure injury was performed by the researchers and the risks that may cause pressure injury in surgical patients were questioned (Engels et al. 2016; Rao et al. 2016; Kim et al. 2018; Konateke 2021; Tang et al. 2021; de Oliveira et al. 2017; Peixoto et al. 2019; Haisley et al. 2020; Guzman et al. 2021; Köksel 2023). Previously developed, valid and reliable scales on the subject were analysed. Preoperative, intraoperative and postoperative risk factors that contribute to the development of surgery‐related pressure injury were determined. This pool consisted of 47 risk factors. The item pool was evaluated by the researchers and a preliminary scale was prepared including age, BMI, ASA score, anaesthesia type, operative position, operative time, amount of bleeding, body temperature (intraoperative and postoperative), blood pressure change, use of support surface and comorbidities. After the scoring system of the preliminary draft scale was determined, it was sent to the experts for their opinions.

#### 3.6.2 Getting Expert Opinions

In order to determine the content validity of the draft scale, the content validity index (CVI) developed by Waltz and Bausel in 1981 was calculated. The “Expert Opinion Evaluation Form” was sent to 22 people including 12 experts who were academicians in the field of surgical nursing, 1 internal medicine physician, 2 anesthesiologists and 7 operating room nurses (specialist or certified nurse) who are experts in the field of surgical nursing. In line with the expert opinions, the Content Validity Index (CVI) was calculated by the researchers and the scale items were revised in line with the recommendations. After the necessary corrections were made in the scale, the pre‐application was started.

#### 3.6.3 Performing the Preliminary Application

Preliminary application was performed in 10 patients who met the inclusion criteria. Patients included in the pre‐application were not included in the sample. Scale items were evaluated for applicability and comprehensibility after the pre‐application. Items that were difficult to evaluate and understand were revised or removed by the researchers and the scale was finalised. During the pre‐application, it was determined that blood albumin levels were not routinely checked in every patient during the preoperative preparation phase in the hospital where the study was conducted. Therefore, the “hypoalbuminemia (< 3 mg/dL)” in the “comorbidity” was removed from the scale.

#### 3.6.4 Data Collection

Research data were collected after ethics committee approval and institutional permissions were obtained. The researchers collected data from 200 patients and the observant operating room nurse collected data from 30 patients. When the patients to be operated on came to the operating room, they were informed about the study in the preoperative room and their written and verbal consent was obtained. Then, the information in the “Patient Descriptive Characteristics Form” was filled in by asking the patient. Patients included in the study did not receive premedication/sedation before coming to the preop room. The study started with 202 patients and 2 patients were excluded from the study. The physician recommended that the patient should not be moved after the surgery according to his/her health condition. Therefore, it could not be checked whether the 2 patients developed pressure injury.

The patient was observed throughout the operation and data collection tools were completed. SURPIRAS data were collected from the patient file (age, BMI, ASA score, type of anaesthesia, comorbidity) and intraoperative observation (wetness in areas under pressure, use of support surfaces, use of heating devices, duration of surgery, amount of bleeding). Body temperature was monitored to determine the “hypothermia status” in the scale. For this purpose, the patient's body temperature was measured with a tympanic thermometer before and every 15 min after induction of anaesthesia. If the patient's body temperature was below 36.0°C during this period, the relevant field in the scale was marked as “hypothermia‐yes”. While the data were collected, the humidity and temperature in the operating room were within normal limits in accordance with evidence‐based guideline recommendations.

After the patient was monitored in the operating room and before the patient was awakened, blood pressure was recorded and the “blood pressure change” field on the scale was completed. If the patient did not develop hypotension during the operation, “1 point” was given in the blood pressure change field and “2 points” was added to the total score of the scale.

Since it is known that surgery‐related pressure injury develops in the first 72 h postoperatively, patients were evaluated with the “Postoperative Patient Evaluation Form” in the first 72 h postoperatively. The first postoperative evaluation of the patient was performed in the first 24 h. Evaluation was performed between the 25th–48th hours and 49th–72nd hours until the patient was discharged. For patients hospitalised longer than 72 h, follow‐up was ended at 72 h.

### 3.7 Statistical Analysis

Data were evaluated with SPSS 22.0 and MedCalc 18.11 programs. Number, percentage and mean ± standard deviation were calculated for descriptive data. The validity of the scale was evaluated by content validity and criterion‐related validity. For content validity, the CVI was calculated, and for criterion‐related validity, the correlation coefficient was determined. The reliability of the developed scale was evaluated with the intraclass correlation coefficient (ICC, Intra‐Class Correlation Coefficient) and Cohen kappa, which are used to determine interobserver agreement. ROC (Receiver Operating Characteristic) analysis was used to determine the cut‐off point of the scale. Sensitivity and specificity values of the cut‐off point were calculated. Independent samples t test or analysis of variance was used to compare the scores obtained from the developed scale according to postoperative patient evaluation parameters. The relationship between the scales compared in the study was evaluated with Pearson correlation coefficient. For the analyses, *p* < 0.05 was accepted as significant.

### 3.8 Ethical Aspects of the Study

Approval for the study was obtained from Gaziantep University Clinical Research Ethics Committee (Decision No: 2023/235, Date: 20.09.2023). Permission was obtained from the university hospital where the study was conducted and the authors of the scales used in the study. The purpose of the study was explained to the patients participating in the study and their written and verbal consent was obtained with the “Informed Voluntary Consent Form”. The study was conducted in accordance with the Declaration of Helsinki.

## 4 Results

### 4.1 Findings Related to Descriptive Characteristics of Patients

When the demographic characteristics of the patients were analysed, it was determined that the mean age was 49.68 ± 16.5 years, 52.5% were female, 65.5% were literate/primary school graduates, 85% were married, 66.5% had no chronic disease and 65.5% were non‐smokers.

In the preoperative evaluation of the operated patients, it was determined that 99% had no pain other than the operation site, 98% had normal skin temperature (not hot or cold), 97.5% had normal skin colour and skin hydration and 99.5% had no oedema. Blood transfusion was not administered intraoperatively in 98% of the patients, and the position was not changed in 98.5%. 67% of the patients underwent surgery in the supine position. In addition, prone, lateral, semi‐Fowler, Fowler, trendelenburg, reverse trendelenburg and lithotomy positions were also used as surgical positions. Position changes were made only in 3 patients, and the position changes were made by surgical team members during the surgery. These patients were brought from the supine position to the semi‐Fowler and lateral position upon the surgeon's request, an average of 75 ± 39.69 min after the start of the surgery. A support surface was used in 62.5% of the patients to reduce pressure on the operating table. The support surface used was viscoelastic in 93.6% of the cases and gel in 6.4% of the cases.

In the postoperative evaluation of the patients, it was determined that 99% of the patients had no pain outside the surgical site, 82.5% had normal skin temperature (not hot or cold), 83% had normal skin colour and skin hydration. Postoperative oedema did not develop in 98% of the patients.

There was no finding of postoperative nerve injury due to pressure and only one patient had a finding of eye injury (stinging sensation). This patient was operating in the prone position. Postoperative pressure injury developed in 20.5% of the patients. In 92.7% of the patients with pressure injury, the pressure injury was seen as Stage 1. 47.5% of pressure injuries occurred in the coccyx/sacrum (Table 1).

**TABLE 1:** Distribution of postoperative pressure injuries in patients (*n* = 200).

Features	*n*	%
Postoperative finding of nerve injury		
Yes	0	—
No	200	100
Postoperative evidence of eye injury		
Yes	1	0.5
No	199	99.5
If yes; symptom		
Stinging sensation	1	100
Postoperative pressure injury status		
Yes	41	20.5
No	159	79.5
Postoperative pressure injury stage		
Stage 1	38	92.7
Stage 2	2	4.9
Mucosal membrane pressure sore	1	2.4
Postoperative pressure injury locations		
Scapula	12	30.0
Coccyx/sacrum	19	47.5
Arm	1	2.5
Hip	1	2.5
Ear	1	2.5
Jaw	3	7.5
Chest/breast	3	7.5

67% of the patients were operated in the supine position and 5.5% in the Fowler/semi‐Fowler position. The number of patients operated in these two positions constitutes the majority of the sample. In both positions, the sacrum region is an area under pressure. Therefore, pressure injury is more developed in this area. In all patients who developed a pressure injury, the pressure injury occurred at one of the pressure points that remained under pressure in the given surgical position. No injuries occurred at any pressure point unrelated to the given surgical position.

### 4.2 Findings Related to Descriptive Characteristics of the Scales

The descriptive characteristics of the developed SURPIRAS are given in Table 2.

**TABLE 2:** SURPIRAS descriptive characteristics (*n* = 200).

Surgery‐Related Pressure Injury Risk Assessment Scale		*n*	%
Age	18–39	52	26.0
	40–49	46	23.0
	50–59	35	17.5
	60–69	46	23.0
	70 and above	21	10.5
BMI	18.6–24.9 kg/m^2^ (normal)	60	30.0
	25.0–29.9 kg/m^2^ (overweight)	95	47.5
	30.0–34.9 kg/m^2^ (grade 1 obesity)	29	14.5
	35.0–39.9 kg/m^2^ (grade 2 obesity)	6	3.0
	≤ 18.5 kg/m^2^; ≥ 40 kg/m^2^ (malnutrition or morbid obesity)	10	5.0
ASA score	I	22	11.0
	II	95	47.5
	III	83	41.5
	IV	—	—
	V	—	—
Anaesthesia type	Local	12	6.0
	Peripheral nerve block	3	1.5
	Neuraxial nerve block (spinal/epidural)	24	12.0
	Sedation	13	6.5
	General	148	74.0
Duration of surgery	≤ 2 h	92	46.0
	> 2 h ≤ 3 h	74	37.0
	> 3 h ≤ 4 h	26	13.0
	> 4 h ≤ 5 h	5	2.5
	> 5 h	3	1.5
Amount of bleeding	≤ 200 cc	147	73.5
	201 cc–400 cc	29	14.5
	401 cc–600 cc	12	6.0
	601 cc–800 cc	3	1.5
	> 800 cc	9	4.5
Wetness in areas under pressure	No	160	80.0
	Yes	40	20.0
Position change	Yes	3	1.5
	No	197	98.5
Support surface usage	Yes	125	62.5
	No	75	37.5
Hypothermia development	No	167	83.5
	Yes	33	16.5
Using heating equipment	Yes	99	49.5
	No	101	50.5
Blood pressure change	Normal blood pressure	165	82.5
	Low blood pressure	35	17.5
Comorbidity Hypertension Diabetes Peripheral arterial disease Chronic venous insufficiency Vasculitis Pulmonary/respiratory diseases History of pressure injury Previously diagnosed neuropathy Deep vein thrombosis	No comorbidities	133	66.5
	1 comorbidity	42	21.0
	2 comorbidities	17	8.5
	3 comorbidities	8	4.0

In this study, the risk of pressure injury was evaluated with three different scales. The total scores of the scales used are given in Table 3.

**TABLE 3:** Total scores of pressure injury risk assessment scales.

	Mean ± SD	Median	Minimum	Maximum
SURPIRAS total score	24.63 ± 4.64	24.00	16.00	37.00
Munro scale total score	23.71 ± 4.25	23.00	16.00	37.00
ELPO total score	17.26 ± 2.99	17.00	11.00	28.00

Abbreviations: ELPO, Risk Assessment Scale for Injuries Related to Surgical Position; Munro Scale, Perioperative Pressure Injury Risk Assessment Scale; SURPIRAS, Surgery‐Related Pressure Injury Risk Assessment Scale.

SURPIRAS scale total score showed a statistically significant difference according to the development of postoperative pressure injury (*p* < 0.05). The total score of the scale was higher in those who developed postoperative pressure injury (Table 4).

**TABLE 4:** Comparison of SURPIRAS total score with postoperative pressure injury status (*n* = 200).

SURPIRAS total score	*n*	Mean ± SD	*t*	*p*
Postoperative pressure injury status				
Yes	41	31.81 ± 2.86	17.947	0.001*
No	159	22.77 ± 2.88		

* *p* < 0.05.

### 4.3 Findings Related to the Validity of the Surgery‐Related Pressure Injury Risk Assessment Scale

Content validity, predictive validity as criterion‐dependent validity and equivalent scale validity were used to determine the validity of the SURPIRAS. The CVI for the items of the draft scale was between 0.90–1.00, and the scale mean CVI was calculated as 0.94. For SURPIRAS predictive validity, postoperative pain status (outside the surgical site), evidence of postoperative nerve or eye injury and postoperative pressure injury were determined. SURPIRAS total score showed statistically significant differences according to postoperative skin condition, skin colour, skin hydration, oedema status and pressure injury status (*p* < 0.05). Patients with abnormal postoperative skin condition, skin colour and skin hydration, and patients with oedema had a higher scale total score. Patients who developed postoperative pressure injury had a significantly higher scale total score than patients without pressure injury (*p* < 0.05).

There was a statistically significant, very strong and positive correlation between SURPIRAS and Munro Scale (*p* < 0.05; *r* = 0.927). A statistically significant, strong and positive correlation was found between SURPIRAS and ELPO (*p* < 0.05; *r* = 0.735) (Table 5).

**TABLE 5:** Relationship between the total scores of the scales.

	SURPIRAS total score	Munro total score	ELPO total score
SURPIRAS total score			
*r*	1	0.927	0.735
*p*		0.001*	0.001*
*N*		200	200
Munro total score			
*r*		1	0.661
*p*			0.001*
*N*			200
ELPO total score			
*r*			1
*p*			
*N*			

*Note:* *r*, Pearson's correlation coefficient; *N*, sample.

* *p* < 0.05.

### 4.4 Findings Related to the Reliability of the Surgery‐Related Pressure Injury Risk Assessment Scale

SURPIRAS reliability was determined by independent inter‐observer agreement. For this, the intraclass correlation coefficient (ICC) was calculated. One operating room nurse assessed 30 patients as an observer. ICC = 0.999 (95% Confidence Interval (CI) (0.997–0.999)) for SURPIRAS total score, ICC = 0.998 (95% CI (0.996–0.999)) for Munro Scale total score, and ICC = 1.000 (95% CI (1–1)) for ELPO scale total score.

In the interobserver correlation analysis for the SURPIRAS, the intraclass correlation coefficient was calculated as 0.792 for the item “wetness in areas under pressure” and 1.00 for all other scale items.

Kappa statistics were used between the investigator and the observer in the postoperative patient evaluation. Kappa statistics indicate whether the agreement between the observer and the investigator was due to chance. Accordingly, the Cohen kappa coefficient was calculated as 0.814 for postoperative pressure injury status and 1.00 for pain, skin condition, skin colour, skin hydration, oedema status, sign of nerve injury and eye injury.

### 4.5 Cut‐Off Point Determination Using Munro Scale and ELPO for SURPIRAS

**FIGURE 2:**
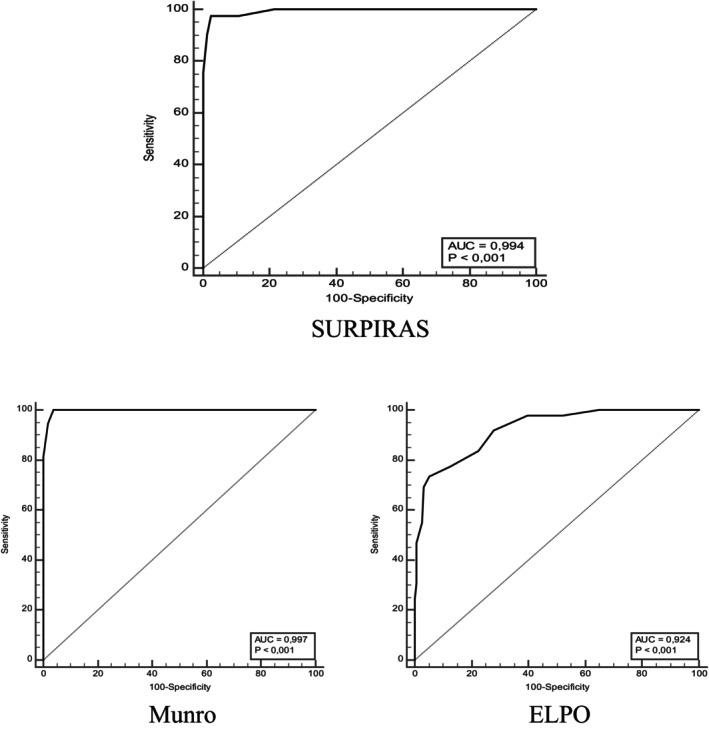
ROC curve.

As a result of the ROC analysis conducted with Munro Scale and ELPO for SURPIRAS, the cut‐off point was determined as 27, and individuals with scores above this value were classified as high risk. The cut‐off point obtained was statistically significant, and the area under the curve (AUC) was determined as 0.994 for SURPIRAS, 0.997 for Munro Scale, and 0.924 for ELPO (Figure 2). In addition, sensitivity and specificity values, and positive and negative predictive values of the cut‐off points of the 3 scales according to the development of pressure ulcers (yes‐no) are given in Table 6.

**TABLE 6:** Results of ROC analysis using Munro scale and ELPO to determine the cut‐off point for SURPIRAS according to pressure injury development.

Scale	Cut‐off point	Sensitivity	95% CI	Specificity	95% CI
SURPIRAS	> 27	97.56	87.1–99.9	97.48	93.7–99.3
Determining the cut‐off point using Munro scale	> 27	100.00	90.7–100.0	96.30	92.1–98.6
Determining the cut‐off point using ELPO	> 27	73.47	58.9–85.1	94.70	89.8–97.7

Abbreviation: CI, confidence interval.

The ROC curve for cut‐off point determination with the SURPIRAS, Munro Scale and ELPO is given in Figure 2.

## 5 Discussion

### 5.1 Discussion of the Analyses Related to the Validity of the Surgery‐Related Pressure Injury Risk Assessment Scale

Content validity shows the adequacy of the scale in covering and collecting the data to be measured. The quality and number of experts to be consulted in content validity is important. While the number of experts is specified as a minimum 3 and a maximum 20 in some sources (Erdogan et al. 2018), it is specified between 5 and 40 by other authors (Lawshe 1975; Ayre and Scally 2014). In this study, 22 experts were consulted.

Many techniques are used to determine content validity. The most frequently used of these are Lawshe and Davis techniques. Davis technique was used in this study. Accordingly, a score of 0.80 and higher than 3 and 4 points given by the experts on each scale item indicates that the content validity is good. Items with a score of 1–2 by the experts should be removed from the scale or reorganised (Erdogan et al. 2018). In this study, the CVI values for each item and the overall scale were above 0.80. This shows that the content validity of the scale is high. However, the scale items that received low scores by the experts were revised and revised. Accordingly, although the “operating room position” item in the draft scale had a CVI value of 0.95 (high), it was stated as an item recommended for revision. In the draft scale sent to the experts, the operating room position item was scored as “Fowler/semi‐Fowler = 1 point”, “lithotomy/trendelenburg/reverse trendelenburg = 2 points”, “lateral = 3 points”, “supine = 4 points”, “prone = 5 points”. In the literature review, studies in the Cochrane database indicate that there is no specific position that is most effective for preventing pressure injury in adults and there is insufficient evidence on the frequency of repositioning. There is insufficient evidence on how different types of positions affect pressure injury (Gillespie et al. 2020, 2021). Therefore, it was determined that it was incorrect to classify the positions as low‐scoring (low risk) or high‐scoring (high risk) in terms of surgery‐related pressure injury. Therefore, the item “surgery position” was removed from the scale and replaced with “position change (yes‐no)”.

Other scale items that were suggested to be revised in line with expert opinions were “body temperature‐operation entry” and “body temperature‐operation exit”. In the draft scale sent to the experts, “body temperature‐operation entry” and “body temperature‐operation exit” items are scored as 36.5°C–37.8°C = 1 point, ‘36.1°C–36.4°C = 2 points’, ‘34.1°C–36.0°C = 3 points’, ‘32.0°C–34.0°C = 4 points’, ‘below 32°C–37.8°C above = 5 points’. Here, it is aimed for the hypothermic patient to receive a high score. However, hypothermia, which is a risk factor associated with surgery‐related pressure injury, is a body temperature below 36°C (Simegn et al. 2021). Therefore, when the patient's body temperature is below 36°C, hypothermia should be accepted and scored only once. Therefore, these items were removed from the scale. In line with expert opinions and the literature, “development of hypothermia (yes = 2 points, no = 1 point)” was added instead of the removed scale item.

Predictive validity shows what the concept to be measured is compared with in real life. A strong correlation between the total score of the scale and the external criterion shows that predictive‐validity is high (Grove et al. 2012; Erdogan et al. 2018). Postoperative development of pressure injury and findings of pain, skin condition, oedema, nerve and eye injury that may affect it are external predictive criteria for the scale. In this study, SURPIRAS total score was found to be significantly higher in patients with abnormal skin condition (hot or cold skin), skin colour and skin hydration, those with oedema and those who developed pressure injury after surgery (*p* < 0.05). A strong positive correlation was found between postoperative pressure injury development and the total score of the scale. This indicates that the scale has predictive validity.

Concurrent validity is the evaluation of the concordance between a previously validated and reliable scale and another scale with the same purpose. The correlation coefficient between the two scales is calculated and a high correlation indicates that there is concurrent validity (Erdogan et al. 2018). To determine the concurrent validity of SURPIRAS, the Munro Scale and ELPO, whose Turkish validity and reliability were previously established, were used to determine the risk of surgery‐related pressure injury. A strong positive relationship was found between SURPIRAS and the Munro Scale and ELPO. This shows that the concurrent validity of the scale is high.

### 5.2 Discussion of the Analyses Related to the Reliability of the Surgery‐Related Pressure Injury Risk Assessment Scale

The reliability of SURPIRAS was determined by independent inter‐observer agreement. The Kappa statistic is used in the calculation of independent inter‐observer agreement analysis to reduce the effect of chance‐based consistency. Kappa takes a value between “+1” and “−1”. “+1” indicates perfect agreement between observers, while “−1” indicates high disagreement between observers. If this value is zero, it is stated that the agreement depends on chance (Hür et al. 2022). In interpreting Cohen kappa values, the agreement levels suggested by Landis and Koch (1977) were used. Accordingly, they are specified as 0.61–0.80 (substantial), 0.81–1.00 (almost perfect). In the study, the Cohen kappa coefficient for pressure ulcer status, one of the postoperative evaluation parameters, was determined as 0.814 and 1.00 for all other parameters. This shows that the agreement between the observer and the researcher is not due to chance. In addition, the fact that the Cohen kappa coefficient is greater than 0.80 in all parameters examined postoperatively shows that the agreement is almost perfect.

To determine the agreement between independent observers, ICC was calculated for the total scale scores. This value > 0.86 indicates that the agreement is clinically significant and excellent (Portney and Watkins 2009; Fleiss 2011). Since the ICC values calculated for SURPIRAS, Munro Scale, and ELPO were found to be high, it was determined that the inter‐observer agreement was at an excellent level.

### 5.3 Limitations

In the operating room where the study was conducted, body temperature is not routinely monitored in the intraoperative period for each patient. Tympanic membrane temperature measurement, which is a non‐invasive method, was used to determine the “development of hypothermia” in the scale. Therefore, ear surgeries were excluded from the study.

In addition, it was determined that albumin levels were not routinely checked in every patient during preoperative preparation. Since albumin levels were not routinely checked, the “Hypoalbuminemia (albumin < 3 mg/dL)” in the comorbidity was excluded from the scale. These are the limitations of the research.

## 6 Conclusion

As a result of this study conducted to test the validity and reliability of SURPIRAS developed by the researchers, SURPIRAS was determined to be a valid and reliable measurement tool for the Turkish population.

SURPIRAS consists of 13 risk factors for surgery‐related pressure injury. The total score of the scale ranges between 13 and 52. The cut‐off point of the scale is 27. Accordingly, patients with a scale total score of 28 and above are defined as high risk. As the total score of the scale increases, the risk of surgery‐related pressure injury increases.

This scale was developed to assess pressure injuries in patients undergoing surgery. Compared to other scales assessing pressure injuries associated with surgery; it does not require much time, is practical, and can be completed by any member of the surgical team. For example; calculating the weight loss in the “weight loss” item on the Munro Scale as a percentage (such as 2 points for weight loss from 7.5% to 9.9%) is not practical when the operating room environment is taken into account and may vary depending on the patient's recall. In addition, it may be difficult to assess expressions such as “slightly moist, very moist” in the “humidity” section. The use of some support surface materials specified as “support surface” in the ELPO is not common in every country. Therefore, the scoring of this section may not be correct. In addition, operating room positions are scored differently in both scales. The differences in risk of these positions may vary according to the surgical operation. For these reasons, it is thought that SURPIRAS will provide a more practical and objective assessment and will contribute significantly to the literature.

## 7 Recommendations

- The use of the Surgery‐Related Pressure Injury Risk Assessment Scale (SURPIRAS) in determining the risk levels of surgery‐related pressure injury in patients aged 18 years and older who will undergo surgery,
- Use of other body temperature measurement methods in ear surgeries that prevent tympanic body temperature measurement.
- Identification of the risk of pressure injury and its use in cost‐effectiveness studies to reduce it.
- Introducing the scale to operating room nurses and supporting its use in the operating rooms of hospitals,
- It is recommended that the scale be translated into different languages and its use should be expanded.

## Author Contributions

S.K. and Ş.İ.G. contributed to conceptualisation, data curation, investigation and writing – original draft; S.K. and Ş.İ.G. contributed to formal analysis and investigation; S.K. contributed to data curation; S.K. and Ş.İ.G. contributed to validation; S.K. and Ş.İ.G. contributed to Software; S.K. and Ş.İ.G. contributed to conceptualisation, supervision and writing – review and editing. All authors have read and agreed to the published version of the manuscript.

## Ethics Statement

Approval for the study was obtained from Gaziantep University Clinical Research Ethics Committee (Decision No: 2023/235, Date: 20.09.2023). Permission was obtained from the university hospital where the study was conducted and the authors of the scales used in the study. The purpose of the study was explained to the patients participating in the study and their written and verbal consent was obtained with the “Informed Voluntary Consent Form”. The study was conducted in accordance with the Declaration of Helsinki.

## Conflicts of Interest

The authors declare no conflicts of interest.

## Data Availability

The data are not publicly available.
